# Supplementary material for: All-trans retinoic acid induces synaptopodin-dependent metaplasticity in mouse dentate granule cells
Source: eLife. 2021 Nov 1;10:e71983. doi: 10.7554/eLife.71983 (PMC8560091; doi:10.7554/eLife.71983)
Supplement: Transparent reporting form [file elife-71983-transrepform1.docx]

***eLife’s* transparent reporting form**

We encourage authors to provide detailed information *within their submission* to facilitate the interpretation and replication of experiments. Authors can upload supporting documentation to indicate the use of appropriate reporting guidelines for health-related research (see [EQUATOR Network](http://www.equator-network.org/%20)), life science research (see the [BioSharing Information Resource](https://biosharing.org/)), or the [ARRIVE guidelines](http://www.plosbiology.org/article/info:doi/10.1371/journal.pbio.1000412) for reporting work involving animal research. Where applicable, authors should refer to any relevant reporting standards documents in this form.

If you have any questions, please consult our Journal Policies and/or contact us: [editorial@elifesciences.org](mailto:editorial@elifesciences.org).

**Sample-size estimation**

- You should state whether an appropriate sample size was computed when the study was being designed
- You should state the statistical method of sample size computation and any required assumptions
- If no explicit power analysis was used, you should describe how you decided what sample (replicate) size (number) to use

Please outline where this information can be found within the submission (e.g., sections or figure legends), or explain why this information doesn’t apply to your submission:

Sample size estimation was determined during the evaluation of animal experiments and approved by the local authorities: University of Freiburg, AZ G-19/152; Faculty of Medicine at the University of Frankfurt, AZ FU/1131 (p. 13, l. 262-264):

Type I error: 0.5%

Estimated effect size prior to experimental assessment: ~1.4 across experiments

Statistical power: 95%

Reevaluation of statistical power was performed during the course of the study.

**Replicates**

- You should report how often each experiment was performed
- You should include a definition of biological versus technical replication
- The data obtained should be provided and sufficient information should be provided to indicate the number of independent biological and/or technical replicates
- If you encountered any outliers, you should describe how these were handled
- Criteria for exclusion/inclusion of data should be clearly stated
- High-throughput sequence data should be uploaded before submission, with a private link for reviewers provided (these are available from both GEO and ArrayExpress)

Please outline where this information can be found within the submission (e.g., sections or figure legends), or explain why this information doesn’t apply to your submission:

Replicates were defined as follows (according to Blainey et al., Nature Methods, 11,879-880(2014)):

One individual mouse: biological replicate

Individual hippocampal slice: technical replicate

One hippocampal tissue sample (RNA-seq): biological replicate

Individual granule cell: biological replicate

Individual dendritic region (EM-analysis): biological replicate

Individual synapse (EM-analysis): biological replicate

Statistics were made on biological replicates only.

All experiments have been replicated in at least 3 independent biological samples.

Figure Legends:

Figure 1: p. 26

Figure 2: p. 26

Figure 3: p. 26

Figure 4: p. 27

Figure 5: p. 27

Figure 6: p. 27

Figure 7: pp. 27, 28

General Inclusion/Exclusion-Criteria:

Pharmacological treatment of animals (p. 13, ll. 273, 274): After injection, no overt behavioral changes were observed. Experiments were performed 3–6 hours after intraperitoneal injection.

RNA-seq analysis (p. 16, ll. 353, 354): All files contained more than 45 M high quality reads having at least a phred quality of 30 ( > 90% of total reads).

Single-cell electrophysiology (p. 15, ll. 311, 312): Series resistance was monitored and recordings were discarded if series resistance reached > 30 MΩ.

Excluded Animals/Data Points:

Exclusion of one Synpo^-/-^ animal during the LTP experiments (incl. justification, p. 19, ll. 413-416):

In these experiments, one Synpo-/- animal in the vehicle-only group was excluded from further analysis, since an insufficient response to increasing stimulus intensities was detected in the input-output curve.

Exclusion of one cell in single-cell electrophysiology (p. 18, ll. 407-409): One individual cell (control group, ventral hippocampus) was excluded from the analysis of intrinsic membrane properties, since the membrane patch lost its integrity during the recordings.

Data points outside the axis limits were reported in the figure legends.

Data Availability:

Data and statistical analysis (Software: GraphPad Prism) are accessible through the following link (Dryad platform): https://datadryad.org/stash/share/oyI2XMauTOtGebtpvtRd8Em8RY0ChLtJox1T5mpQ00Q. RNA sequencing data are accessible from the galaxy web platform via the following link: https://usegalaxy.eu/u/maximilian.lenz/h/transcriptome-analysisatra-6h-vs-controlhippocampus.

**Statistical reporting**

- Statistical analysis methods should be described and justified
- Raw data should be presented in figures whenever informative to do so (typically when N per group is less than 10)
- For each experiment, you should identify the statistical tests used, exact values of N, definitions of center, methods of multiple test correction, and dispersion and precision measures (e.g., mean, median, SD, SEM, confidence intervals; and, for the major substantive results, a measure of effect size (e.g., Pearson's r, Cohen's d)
- Report exact p-values wherever possible alongside the summary statistics and 95% confidence intervals. These should be reported for all key questions and not only when the p-value is less than 0.05.

Please outline where this information can be found within the submission (e.g., sections or figure legends), or explain why this information doesn’t apply to your submission:

Description of statistical tests (p. 19; ll. 424-432):

Data were statistically evaluated using GraphPad Prism 7 (GraphPad software, USA). Statistical comparisons were made using the non-parametric Mann-Whitney test. For statistical comparison of XY-plots in whole-cell patch-clamp recordings, we used an RM two-way ANOVA test (repeated measurements/analysis) with Sidak’s multiple comparisons. Statistical analysis of fEPSP slope data was performed using the Mann-Whitney test for the three terminal data points. p-values smaller 0.05 were considered a significant difference. In the text and figures, values represent mean ± standard error of the mean (s.e.m.). Statistical significance in XY-plots is indicated in the figure panel. U-values were provided for significant results only. *, p < 0.05; ***, p < 0.001; ‘ns’, not significant differences.

Statistical evaluation of transcriptome analysis (p. 18, ll. 396-399): Statistical evaluation was performed using DESeq2 (Galaxy version 2.11.40.6+galaxy1) with treatment as the primary factor that might affect gene expression. Genes were considered as differentially expressed if the adjusted p-value was < 0.05. Heat maps were generated based on z-scores of the normalized count table.

All figure legends contain information as follows:

Figure 1 (p. 26): Individual data points are indicated by gray dots. Values represent mean ± s.e.m. (*, p < 0.05; ns, non-significant difference).

Figure 2 (p. 26): Individual data points are indicated by gray dots. Values represent mean ± s.e.m. (ns, non-significant difference).

Figure 3 (p. 26): Individual data points are indicated by gray dots. Values represent mean ± s.e.m. (ns, non-significant difference).

Figure 4 (p. 27): Individual data points are indicated by gray dots. Values represent mean ± s.e.m. (ns, non-significant difference).

Figure 5 (p. 27): DESeq2-Analysis indicates the differential expression of 29 genes with a moderate │log2FC│ < 1 (visualization by MA plot).

Figure 6 (p. 27): Individual data points are indicated by colored dots. Values represent mean ± s.e.m. (***, p < 0.001; ns, non-significant difference).

Figure 7 (p. 28): Values represent mean ± s.e.m. (*, p < 0.05; ns, non-significant difference).

Values of N were reported in the figure legends along with the respective statistical tests.

(For large datasets, or papers with a very large number of statistical tests, you may upload a single table file with tests, Ns, etc., with reference to sections in the manuscript.)

**Group allocation**

- Indicate how samples were allocated into experimental groups (in the case of clinical studies, please specify allocation to treatment method); if randomization was used, please also state if restricted randomization was applied
- Indicate if masking was used during group allocation, data collection and/or data analysis

Please outline where this information can be found within the submission (e.g., sections or figure legends), or explain why this information doesn’t apply to your submission:

Group Allocation:

Mice were randomly assigned to the treatment groups.

Blinding:

If no automated analysis was applied, analysis was performed and validated by investigators blind to experimental conditions (p. 19; ll. 417-418): Electron microscopy images were analyzed and cross-checked by five investigators blind to experimental conditions.

**Additional data files (“source data”)**

- We encourage you to upload relevant additional data files, such as numerical data that are represented as a graph in a figure, or as a summary table
- Where provided, these should be in the most useful format, and they can be uploaded as “Source data” files linked to a main figure or table
- Include model definition files including the full list of parameters used
- Include code used for data analysis (e.g., R, MatLab)
- Avoid stating that data files are “available upon request”

Please indicate the figures or tables for which source data files have been provided:

Data and statistical analysis for all figures (Software: GraphPad Prism) are accessible through the following link (Dryad platform): https://datadryad.org/stash/share/oyI2XMauTOtGebtpvtRd8Em8RY0ChLtJox1T5mpQ00Q.

Custom MATLAB scripts for fEPSP population spike analysis are accessible through the following link (github.com): https://github.com/juliamuellerleile/population-spike-analysis.

RNA sequencing data are accessible from the galaxy web platform via the following link: https://usegalaxy.eu/u/maximilian.lenz/h/transcriptome-analysisatra-6h-vs-controlhippocampus.
